# Comparison of Three Pulpotomy Agents in Primary Molars: A Randomised Clinical Trial

**Published:** 2011-02-15

**Authors:** Elham Farrokh Gisoure

**Affiliations:** 1. Department of Pediatric, Oral and Dental Diseases Research Center/Kerman University of Medical Sciences, Kerman, Iran.

**Keywords:** Electrosurgery, Ferric Sulfate, Formocresol, Primary tooth, Pulpotomy

## Abstract

**INTRODUCTION:**

Pulpotomy is an accepted treatment for the management of cariously exposed pulps in symptom free primary molars. The purpose of this study was to compare the clinical and radiological outcomes of three different single-visit vital pulp therapies including pulpotomy with electrosurgery (ES), formocresol (FC) and ferric sulfate (FS) in cariously exposed primary molar teeth.

**MATERIALS AND METHODS:**

Seventy-six patients, 5-10 years old, were enrolled in this clinical investigation. One primary molar tooth of each patient was selected for this study. Samples were randomly divided into three groups. Teeth were treated with FC in group 1 (i.e. control group) (n=24), FS in group 2 (n=28), and ES in group 3 (n=24) using standardized pulpotomy procedures. All treated teeth were clinically and radiographically evaluated after 6 and 9 months. Statistical analysis was performed using Fishers exact test.

**RESULTS:**

The overall success rate in groups 1, 2 and 3 was 87.5%, 82.1% and 83.3%, respectively. Favorable clinical and radiological success rates of FS and ES pulpotomy was observed which was comparable to FC.

**CONCLUSION:**

FS and ES can be considered alternative materials for the pulpotomy of primary molars.

## INTRODUCTION

Pulpotomy is one of the common treatments for of cariously exposed pulps in symptom free primary teeth; the procedure helps to maintain the integrity of primary teeth that have inflammation limited to coronal pulp. The main goal is to preserve the radicular pulp, maintain vitality and ultimately to retain the tooth [1][2]. This treatment can be performed using different techniques including electrosurgery (ES) [3], Er: YAG Laser [4] or by dressing using different materials such as formocresol (FC) [5], calcium hydroxide [6], enriched collagen solution [7], ferric sulfate (FS) [8]. Many other techniques have been suggested [9]; an extensive systemic review could not conclusively provide evidence for the most appropriate technique [1].

FC was firstly introduced by Sweet with a 97% success rate [10]; this material has been considered as the gold standard [11]. FC has been the most commonly used pulp-dressing material for pulpotomy of primary molars during the past six decades [12]; some significant disadvantages e.g. its cytotoxicity, potential mutagenicity [13] and immune sensitization [12] have been a cause of concerns. Clinicians prefer use to alternative methods which are more bio and tissue compatible.

FS is a coagulative and haemostatic agent. Fei et al. reported the application of ferric sulfate in pulpotomized human primary molars with clinical and radiographic success rates of 100% and 97%, respectively [8] No concerns about toxic or harmful effects of ferric sulfate have been recorded in dental literature [14].

ES as a non pharmacological pulpotomy technique that has been well-documented and has proven to have great merits [15]. ES leads to good visualization and homeostasis and is less time consuming than the FC approach [16]. Dean and colleagues did not find any significant difference between the success rates for the electrosurgical and FC pulpotomy techniques [3]. Rivera et al. evaluated postoperative clinical and X-ray film findings from 80 molars after FC and ES vital pulpotomy. They did not find any significant difference between the two techniques after six-months follow up [17].

The purpose of this clinical trial was to compare the clinical and radiographic success of FC, FS and electrosurgical pulpotomy used for pulpotomy of human primary molar teeth requiring vital pulp therapy secondary to carious involvement.

## MATERIALS AND METHODS

Seventy-six patients with the age range of 5-10 years (mean age: 6±1.6) were selected from the patients referred to the pediatric department of Kerman faculty of Dentistry after ethical approval and informed parental consent. All patients had normal physical growth, no systemic disease and were cooperative with at least one symptom free carious primary molar. Inclusion criteria for studied teeth were a) carious exposure of the vital pulp with no symptom, b) no clinical or radiographic evidence of pulpal degeneration and c) restorable coronal caries. Informed consent was obtained from children’s parents or carers. Clinical exclusion criteria were composed of tenderness to percussion, swelling, fistulation, spontaneous pain, and pathologic mobility. Radiographic exclusion criteria were composed of internal or external resorption, widening of periodontal ligament space, and physiologic resorption more than one third of the tooth root. Recent preoperative radiographs were taken from all patients.

### Clinical procedure:

After application of local anesthesia with 5% Xilocaine spray and 2% lidocaine injection, quadrant isolation was performed with rubber dam; and dental caries were removed with a high speed carbide fissure bur. Following pulpal exposure, the superficial pulp was removed with a low speed carbide round bur no.2 (SS white; NJ, USA) and then the whole coronal pulp was amputated with spoon excavator. Samples were assigned randomly to one of the three treatment groups. FC made up the control group in group 1 (n=24), group 2 (n=28) consisted of ferric sulfate, and ES technique was used in group 3 (n=24).

In group 1, the pulp chamber was flushed with 5cc sterile saline and was then dried with sterile cotton pellets. For hemostasis, wet sterile cotton pellets were used. Sterile cotton pellets were saturated with FC and placed in cleaned pulp chamber for 5 minutes. Subsequently, the pulp chamber was dried with a cotton pellet.

In group 2 (FS), the pulp chamber was flushed with 5cc sterile saline and dried with dry sterile cotton pellets. FS was used by the aid of a cotton pellet in canal orifices. Hemostasis was obtained after 10 to 30 seconds, and then blood clots were removed [18].

In group 3 (ES), series of sterile cotton pellets saturated with saline were put in the pulp chamber to obtain hemostasis. Then, the cotton pellets were removed and ES dental U shaped electrode (Colten/Whaledent, Perfect Tissue Contouring, Model No. S7230, USA, System, TCS) was immediately used for tissue coagulation. The ES unit was set at 45-50% power (13.5-15 watts). The electrical current was placed into the pulp for 1 second. This procedure was repeated up to 3 times on each pulpal orifice, until brown appearance was observed in the tissue [16]. In all study groups, zinc oxide-eugenol was placed directly on the radicular pulp stump and the teeth were restored with stainless steel crowns.

Patients were recalled after 6 and 9 months for clinical and radiographic evaluation by a blinded examiner. Clinical success was defined as the absence of spontaneous pain, chronic or acute abscess, fistula or excessive mobility. Radio-graphic success was defined as the presence of a normal periodontal ligament space, absence of furcal radiolucency, pathologic root resorption or root canal calcification.

Statistical analysis was performed using SPSS version 15 software. Statistical significant was defined as P<0.05 and dichotomous variables were compared using Fishers exact test.

## RESULTS

**Figure 1:**
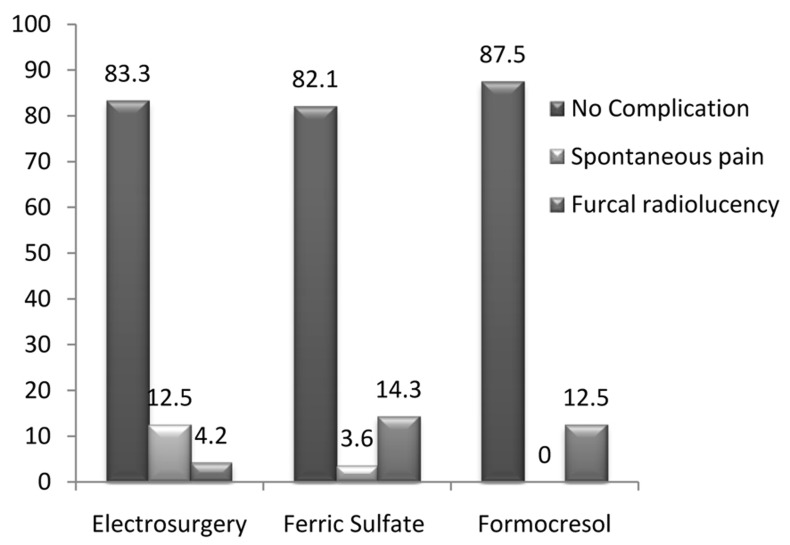
Clinical and radiographic findings in three groups

Our findings showed there were no statistically significant differences between the success rates of the 3 groups (P>0.05). Figure 1 shows the clinical and radiographic status of teeth in study groups after 6 and 9 months.

One tooth in group 2 and three teeth in group 3 showed spontaneous pain during the two intervals. Clinical success rates in groups 1 to 3 were 100%, 96.4%, and 87.5% respectively (Table 1).

**Table 1:** Clinical and radiographic success rate in different groups after 6 and 9 months

**Success rate**	**Electrosurgery**	**Ferric sulfate**	**Formocresol**
**Clinical (no pain)**	21 (87.5%)	27 (96.4%)	24 (100%)
**Radiographic (no furcal radiolucency)**	23 (95.8%)	24 (85.7%)	21 (87.5%)

Radiographic success rates at the 6 to 9 months interval demonstrated three teeth in the control group, four teeth in the second group and one tooth in the third group with furcal radiolucency. That is, success rates were 87.5%, 85.7% and 95.8% for groups 1 to 3, respectively (Table 1).

## DISCUSSION

This study evaluated the clinical outcome of pulpotomy of primary teeth with three different methods.

FC was an extremely popular medicament for pulpotomy of primary teeth. Vital pulpotomy teaching protocols’ in the UK and Ireland indicate that FC is becoming less popular [2]. Concerns about FC safety have been published in dental and medical literature for the past 30 years and have led clinicians to use alternative methods that have more tissue compatibility [1][2][10][11][12].

No correlation between FC pulpotomies and cancer has been demonstrated and therefore FC is still concerned as the gold standard for pulpotomy studies [1][11][12]. The results of this study revealed that the clinical and radiographic success rate of FC were 100% and 87.5% respectively; a value similar those of a previous study [2]; although this success rate was different to those of Waterhouse et al. and Huth et al. who achieved lower success rates [19][20].

Of the failed cases in group 1 (control group), three cases exhibited furcal radiolucency. The failure of pulpotomy treatment in primary molars has been attributed to several factors one of which is clinical errors in diagnosis and selection of primary teeth. For example, chronically inflamed radicular pulps were believed to be non inflamed [2][19].

In group 2, one case exhibited spontaneous pain and four cases showed furcal radiolucency. Clinical and radiographic success rates in this group were 96.4 and 85.7% respectively; which is in parallel with previous study by Burnett and Walker [21]. However, this rate was lower than that reported by others [18][20]. The differences could be attributed to the dissimilar techniques and duration of study.

In group 3, three cases exhibited spontaneous pain and one case showed furcal radiolucency. Clinical and radiographic success rate were 87.5% and 95.8%, respectively; concurring with other studies [3][16]. Our findings were similar to those of Dean et al. that demonstrated the clinical and radiographic success rates for electrosurgical pulpotomy to be comparable to those of FC pulpotomy [3]. However, they differed from another study; this could be attributed to the differences in the applied techniques [22].

Comparable outcomes for electrosurgical, FC and FS pulpotomies of human primary molar teeth during 9-months follow up were shown. There were no significant differences between three groups (experimental and controls). Surveys with larger sample size are needed to clarify any possible differences and provide a more accurate picture.

Success or failure of pulpotomy treatment depends upon an accurate diagnosis. However, FC has proven to be a more forgivable technique that helps to retain primary teeth even with chronic, silent inflammation. On the other hand, pulpotomy with ES appears to require more sensitive diagnosis. However, electrosurgical procedure has two distinct advantages: it is a swifter and drug free procedure with no known undesirable systemic effects [16].

Since FC was known to cause toxicity, immune sensitization, mutagenic and chromosomal aberrations, the safety of this material is questionable [18][23]. The electrosurgical pulpotomy has become more common, due to its non pharmacological nature, ease in use and favorable results [11]. FS has also been used with good haemostatic effects [12] and no reported adverse effects.

## CONCLUSION

The results of our study indicate that the FS and electrosurgical pulpotomy appears comparable to the FC pulpotomy for human primary molars. Further studies are needed to evaluate the histological effect of these methods as well as compare these methods to pulpotomy with new bioregenerative materials. Moreover, other factors that affect the success of the pulpotomy such as coronal seal need to be analyzed.
